# Supplementary material for: Astrocyte Subtype-Specific Expression of the Sodium-Coupled Citrate Transporter SLC13A5 and Citrate Metabolism Genes Across Alzheimer’s Disease Pseudoprogression: A Single-Nucleus RNA Sequencing Analysis of the Human Middle Temporal Gyrus
Source: Curr Issues Mol Biol. 2026 Jul 5;48(7):691. doi: 10.3390/cimb48070691 (PMC13407191; doi:10.3390/cimb48070691)
Supplement: Supplementary file 1 [file cimb-48-00691-s001.zip › Supplementary_files_cimb_final/NEC03V02D0526_MDPI_Supp.pdf]

Supplementary Materials: Astrocyte subtype-specific expression of the sodium-coupled citrate transporter SLC13A5 and citrate metabolism genes across Alzheimer's disease pseudoprogression: a single-nucleus RNA sequencing analysis of the human middle temporal gyrus

Patrícia Fernanda Schuck[1], Gustavo da Costa Ferreira[1], Hércules Rezende Freitas[2]

1. Supplementary Tables

1.1. Table S1. Cell-level Spearman correlations.
1.2. Table S2. Donor-level Spearman correlations.
1.3. Table S3. Neuropathological correlations for all eight citrate metabolism genes.
1.4. Table S4. Reactive astrocyte marker comparison: Astro 2 vs. Astro 3.
1.5. Table S5. SLC13A5 pseudoprogression correlations per astrocyte supertype.
1.6. Table S6. Segmented regression of SLC13A5 astrocyte prevalence across pseudoprogression.

2. Supplementary Figures

2.1. Figure S1. Sex-stratified expression trajectories across pseudoprogression.
2.2. Figure S2. Cognitive status comparison across all eight citrate metabolism genes.
2.3. Figure S3. SLC13A5 trajectory breakpoint and per-supertype pseudoprogression correlations.
2.4. Figure S4. Braak stage and APOE4 associations across all eight citrate metabolism genes.

Table S1. Supplementary Table 1. Cell-level Spearman correlations (pseudoprogression vs. binary expression, astrocyte nuclei).

Gene	Spearman rho	p-value	FDR	Significance	n nuclei
SLC13A5	-0.008	0.0449	0.0609	ns	67419
SLC13A3	-0.080	0.0000	0.0000	***	67419
SLC25A1	-0.003	0.4890	0.4890	ns	67419
ACLY	-0.006	0.1160	0.1330	ns	67419
ACO1	-0.059	0.0000	0.0000	***	67419
ACO2	-0.029	0.0000	0.0000	***	67419
IDH1	-0.008	0.0456	0.0609	ns	67419
IDH2	-0.032	0.0000	0.0000	***	67419

Table S2. Supplementary Table 2. Donor-level Spearman correlations (mean pseudoprogression vs. bulk astrocyte prevalence, n = 84 donors).

Gene	Spearman rho	p-value	FDR	Significance	n donors
SLC13A5	-0.042	0.704000	0.78600	ns	84
SLC13A3	-0.362	0.000723	0.00578	**	84
SLC25A1	-0.073	0.507000	0.78600	ns	84
ACLY	-0.050	0.652000	0.78600	ns	84
ACO1	-0.322	0.002770	0.01110	*	84
ACO2	-0.258	0.017700	0.04710	*	84
IDH1	-0.030	0.786000	0.78600	ns	84
IDH2	-0.170	0.122000	0.24500	ns	84

Table S3. Supplementary Table 3. Neuropathological correlations for citrate gene prevalences. Braak correlations use donor-level prevalence across all astrocytes (n = 84 donors). Thal and CERAD correlations are Astro 2-specific (SLC13A5, SLC13A3, ACO1, ACO2 only).

Gene	Pathology measure	Spearman rho	p-value	FDR	Sig.	n donors
ACLY	Braak stage	-0.086	4.38e-01	0.438000	ns	84
ACO1	Braak stage	-0.430	4.51e-05	0.000361	***	84
ACO1	CERAD score	-0.349	1.13e-03	0.005760	**	84
ACO1	Thal	-0.307	4.49e-03	0.008970	**	84
ACO2	Braak stage	-0.198	7.05e-02	0.113000	ns	84
ACO2	CERAD score	-0.076	4.92e-01	0.492000	ns	84
ACO2	Thal	-0.156	1.57e-01	0.179000	ns	84
IDH1	Braak stage	-0.212	5.29e-02	0.106000	ns	84
IDH2	Braak stage	-0.310	4.17e-03	0.016100	*	84
SLC13A3	Braak stage	-0.297	6.04e-03	0.016100	*	84
SLC13A3	CERAD score	-0.342	1.44e-03	0.005760	**	84
SLC13A3	Thal	-0.310	4.14e-03	0.008970	**	84
SLC13A5	Braak stage	-0.094	3.95e-01	0.438000	ns	84
SLC13A5	CERAD score	-0.161	1.42e-01	0.179000	ns	84
SLC13A5	Thal	-0.241	2.73e-02	0.043700	*	84
SLC25A1	Braak stage	-0.091	4.08e-01	0.438000	ns	84

Table S4. Supplementary Table 4. Wilcoxon rank-sum comparison of reactive astrocyte marker prevalences between Astro 2 and Astro 3 supertypes (astrocyte nuclei, BH-corrected).

Marker	Prevalence Astro 2	Prevalence Astro 3	log2FC (A2/A3)	p-value	FDR	Sig.
GFAP	0.226	0.252	-0.16	0.000	0.000	***
VIM	0.119	0.095	0.33	0.000	0.000	***
C3	0.016	0.104	-2.74	0.000	0.000	***
ALDH1L1	0.256	0.210	0.28	0.000	0.000	***
AQP4	0.255	0.242	0.07	0.363	0.415	ns
S100B	0.114	0.159	-0.49	0.000	0.000	***
CD44	0.055	0.233	-2.07	0.000	0.000	***
LCN2	0.000	0.000	Inf	0.517	0.517	ns

Table S5. Supplementary Table 5. SLC13A5 Spearman correlations with pseudoprogression per astrocyte supertype. Prevalence: binary expression vs. pseudo-score across all nuclei in each supertype (Astro 4 excluded; < 50 expressing cells). Intensity: log1p-transformed counts among SLC13A5-expressing nuclei only (Astro 3 and Astro 4 additionally excluded due to < 100 expressing cells).

Supertype	Measure	n nuclei	n expressing	Spearman rho	p-value	FDR
Astro_1	Intensity (SLC13A5+ only)	399	399	-0.004	9.30e-01	9.30e-01
Astro_1	Prevalence (binary)	5335	399	-0.059	1.55e-05	3.87e-05
Astro_2	Intensity (SLC13A5+ only)	10800	10800	0.042	1.08e-05	4.31e-05
Astro_2	Prevalence (binary)	44919	10800	-0.043	0.00e+00	0.00e+00
Astro_3	Prevalence (binary)	9439	82	0.015	1.34e-01	1.34e-01
Astro_5	Intensity (SLC13A5+ only)	452	452	-0.055	2.45e-01	3.26e-01
Astro_5	Prevalence (binary)	3603	452	-0.037	2.83e-02	3.54e-02
Astro_6	Intensity (SLC13A5+ only)	732	732	0.046	2.10e-01	3.26e-01
Astro_6	Prevalence (binary)	3271	732	0.043	1.29e-02	2.15e-02

Table S6. Supplementary Table 6. Segmented ordinary least-squares regression of SLC13A5 astrocyte prevalence across 20 equal-width pseudoprogression bins (n = 19 bins with data; weighted by nuclei per bin). Davies test evaluates whether the breakpoint improves fit over a linear model.

Parameter	Value
Breakpoint (pseudo-score)	0.5195
Breakpoint SE	0.0992
Slope before breakpoint (per unit pseudo-score)	0.3319
Slope after breakpoint	-0.1940
Adjusted R^2	0.4143
Davies test p-value (breakpoint significance)	0.0392

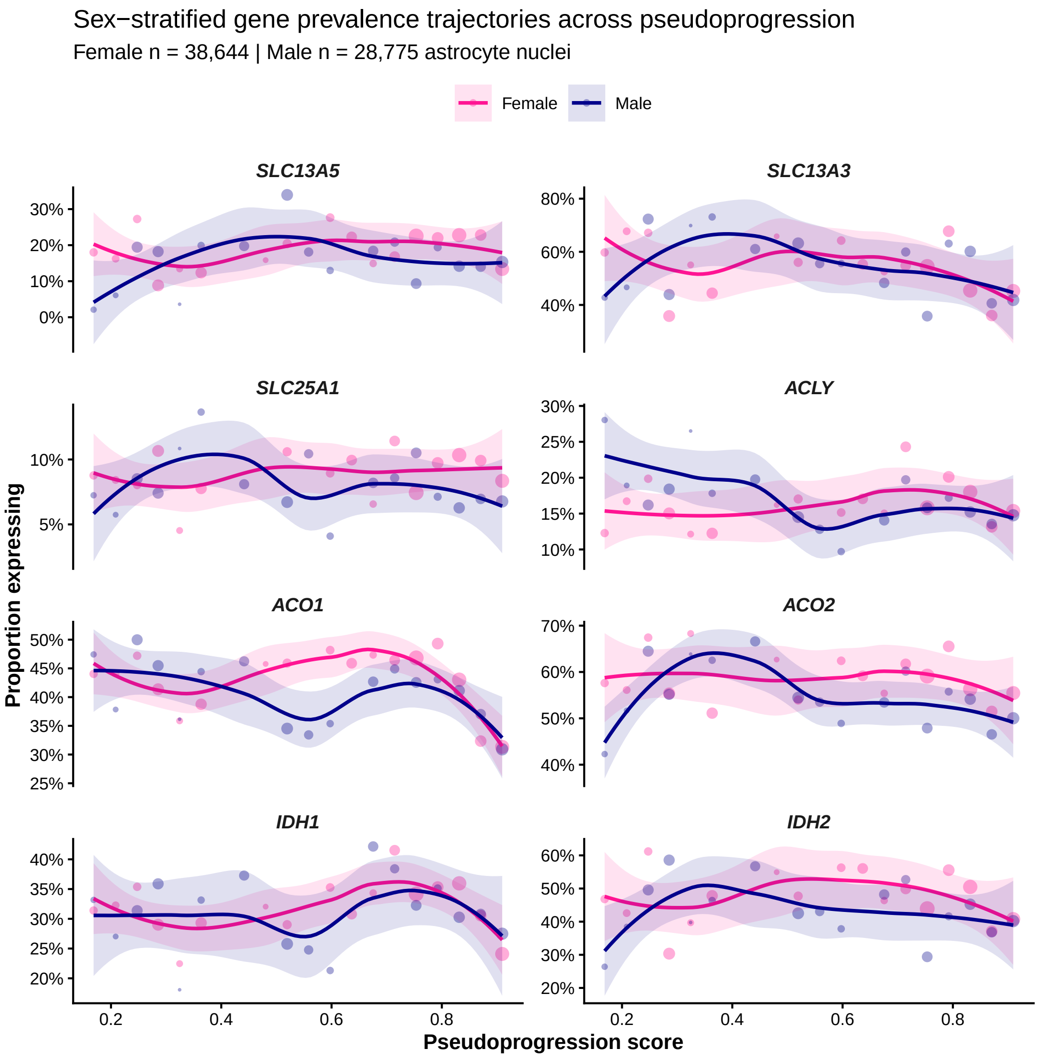

Figure S1. Figure S1. Sex-stratified LOESS-smoothed prevalence trajectories for all eight citrate metabolism genes across the pseudoprogression score (20 equal-width bins; shading = 95% CI). Each point represents one pseudoprogression bin; point size reflects the number of astrocyte nuclei in that bin.

13

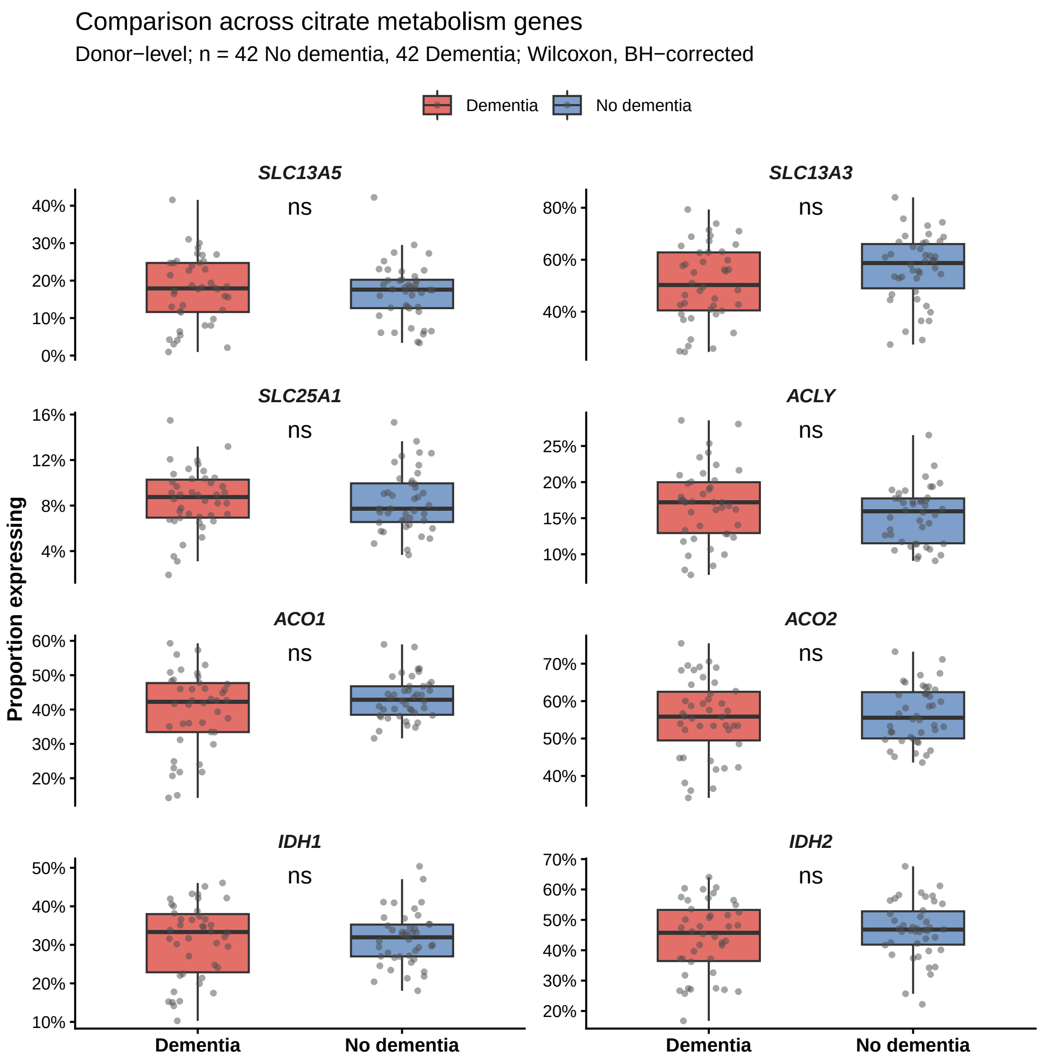

Figure S2. Figure S2. Donor-level prevalence of each citrate metabolism gene stratified by cognitive status (No dementia vs. Dementia; Reference donors excluded). Boxes show median and IQR; significance labels reflect BH-corrected Wilcoxon rank-sum tests (*** FDR < 0.001, ** FDR < 0.01, * FDR < 0.05, ns = not significant).

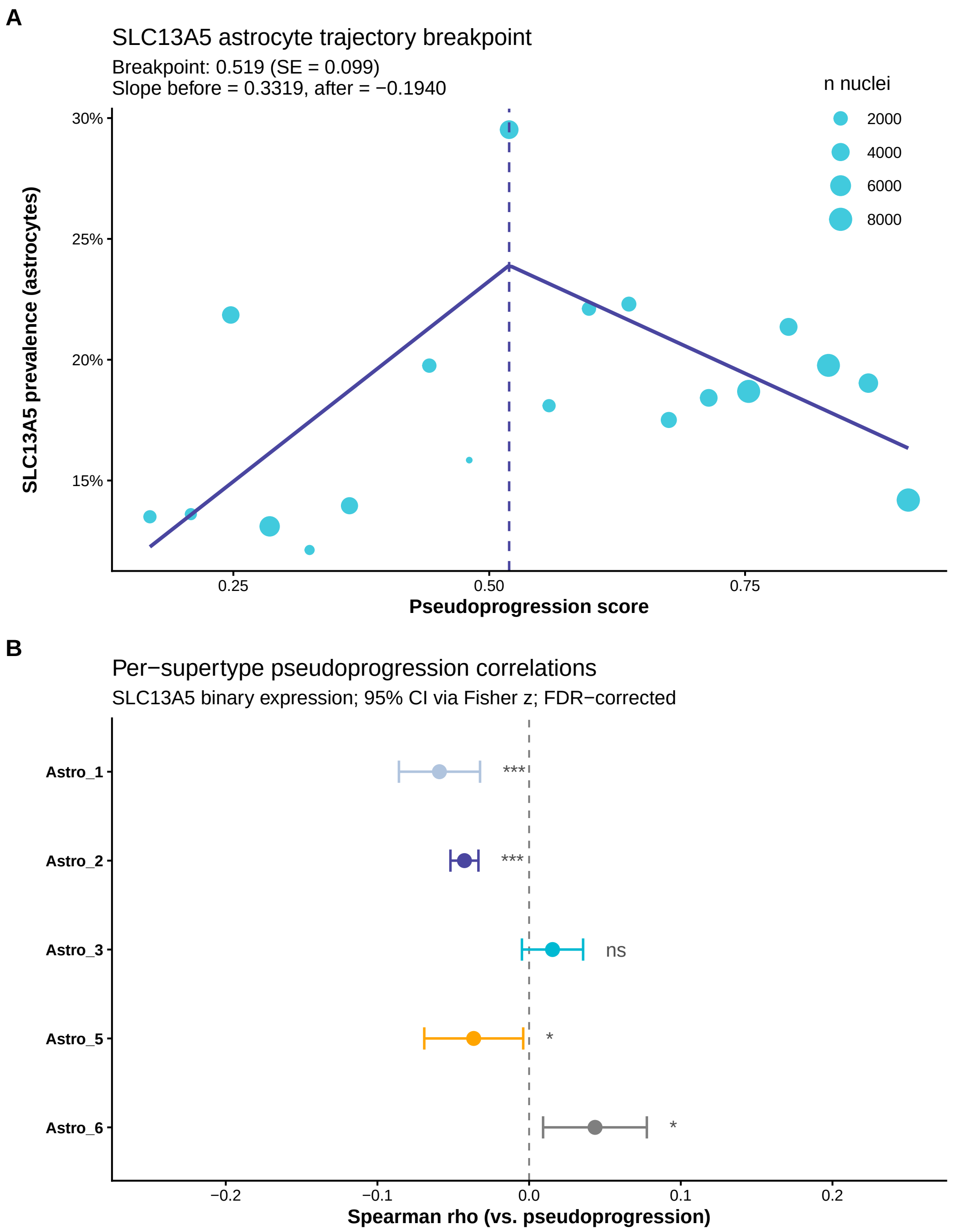

Figure S3. Figure S3. (**A**) Segmented OLS regression of SLC13A5 prevalence across 20 pseudoprogression bins in astrocytes. The dashed vertical line marks the estimated breakpoint; the purple line is the piecewise-linear fit weighted by nuclei per bin. (**B**) Per-supertype Spearman correlations of SLC13A5 binary expression with pseudoprogression score. Error bars = 95% CI via Fisher z-transformation. FDR-corrected significance labels (*** < 0.001, ** < 0.01, * < 0.05, ns).

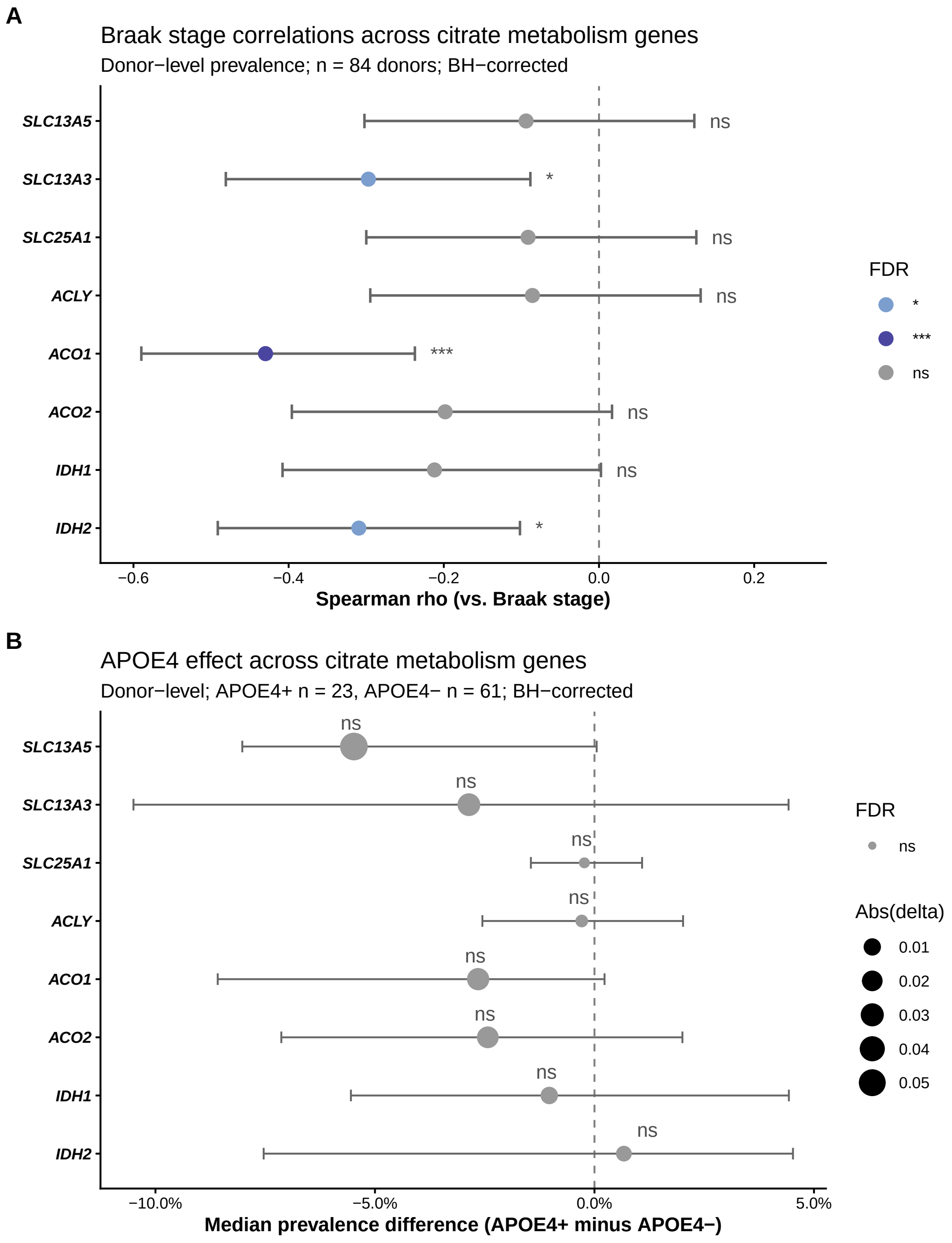

Figure S4. Figure S4. (**A**) Donor-level Spearman correlations between Braak neurofibrillary tangle stage and bulk astrocyte prevalence for each gene (n = 84 donors; 95% CI via Fisher z-transformation; BH-corrected). (**B**) Difference in median donor-level prevalence between APOE4 carriers and non-carriers (Wilcoxon rank-sum; BH-corrected). Point size is proportional to the absolute difference.
