# Supplementary material for: Astrocyte Subtype-Specific Expression of the Sodium-Coupled Citrate Transporter SLC13A5 and Citrate Metabolism Genes Across Alzheimer’s Disease Pseudoprogression: A Single-Nucleus RNA Sequencing Analysis of the Human Middle Temporal Gyrus
Source: Curr Issues Mol Biol. 2026 Jul 5;48(7):691. doi: 10.3390/cimb48070691 (PMC13407191; doi:10.3390/cimb48070691)

LabInfo∫

Health Informatics Laboratory

Contents

1 Title page **1**

1.1 Title . . . 1

1.2 Authors . . . 1

2 Results **2**

2.1 SLC13A5 expression is restricted to astrocytes and concentrated in the Astro 2 supertype . . . 2

2.2 Overall SLC13A5 prevalence is stable across pseudoprogression . . . 4

2.3 Astrocyte subtype composition shifts underlie apparent SLC13A5 stability . . . 4

2.4 Astro 3 is an A1-reactive subtype; Astro 2 is homeostatic . . . 4

2.5 Co-expression of citrate metabolism genes in SLC13A5+ nuclei . . . 5

2.6 SLC13A5 and citrate gene expression correlate with neuropathological burden . . . 7

2.7 APOE4 genotype is associated with lower SLC13A5 prevalence in Astro 2 . . . 7

2.8 SLC13A5 expression does not distinguish cognitively resilient from expected-AD donors 7

3 Supplementary material **10**

3.1 Supplementary Table 1. Cell-level Spearman correlations for all eight citrate metabolism genes across pseudoprogression in astrocytes (n = 67,419 nuclei). . . . 10

3.2 Supplementary Table 2. Donor-level Spearman correlations for all eight genes. . . . 10

3.3 Supplementary Table 3. Neuropathological correlations for all eight citrate metabolism genes. . . . 10

3.4 Supplementary Table 4. Reactive astrocyte marker comparison: Astro 2 vs. Astro 3. 11

3.5 Supplementary Table 5. SLC13A5 expression correlations with pseudoprogression per astrocyte supertype. . . . 12

3.6 Supplementary Table 6. Segmented regression of SLC13A5 astrocyte prevalence across pseudoprogression. . . . 12

3.7 Supplementary Figure S1. Sex-stratified SLC13A5 and citrate metabolism gene expression trajectories across pseudoprogression. . . . 13

3.8 Supplementary Figure S2. Cognitive status comparison across all eight citrate metabolism genes. . . . 14

3.9 Supplementary Figure S3. SLC13A5 astrocyte trajectory breakpoint and per-supertype pseudoprogression correlations. . . . 15

3.10 Supplementary Figure S4. Neuropathological and genetic associations across all eight citrate metabolism genes. . . . 16

Astrocyte subtype-specific expression of the sodium-coupled citrate transporter SLC13A5 and citrate metabolism genes across Alzheimer's disease pseudoprogression: a single-nucleus RNA sequencing analysis of the human middle temporal gyrus

Companion Statistical Report

**Analyst**	Prof. Dr. Hércules R. Freitas
**Laboratory**	Health Informatics Laboratory - LabInfoS
**Version code**	NECS03V02D0526
**Date**	04/07/2026

Schuck, 2026

1 Title page

1.1 Title

Astrocyte subtype-specific expression of the sodium-coupled citrate transporter SLC13A5 and citrate metabolism genes across Alzheimer's disease pseudoprogression: a single-nucleus RNA sequencing analysis of the human middle temporal gyrus

1.2 Authors

Patrícia Fernanda Schuck[1]

Gustavo da Costa Ferreira[1]

Hércules Rezende Freitas[2]

[1]Laboratório de Erros Inatos do Metabolismo, Programa de Bioquímica e Biofísica Celular, Instituto de Bioquímica Médica Leopoldo de Meis, Universidade Federal do Rio de Janeiro, Rio de Janeiro 21941-599, Brazil.

[2]Laboratório de Informática em Saúde (LabInfoS), Departamento de Ciências Médicas Integradas, Faculdade de Ciências Médicas, Universidade do Estado do Rio de Janeiro, Cabo Frio 28905-320, Brazil.

Correspondence: hercules.freitas@uerj.br

2 Results

2.1 SLC13A5 expression is restricted to astrocytes and concentrated in the Astro 2 supertype

Among 1,378,211 nuclei across 24 cell subclasses in the SEA-AD MTG dataset, *SLC13A5* was expressed almost exclusively in astrocytes. The full cellular landscape of the SEA-AD dataset and the spatial distribution of *SLC13A5*-expressing cells are shown in Figure 1A-D. Approximately 20% of astrocyte nuclei were *SLC13A5*-positive. This prevalence was robust across sequencing depth, remaining above 10% even in the lowest library-size quartile of astrocyte nuclei (median 4,054 UMIs per cell; Figure 1E). L5 ET neurons showed 7.7% overall prevalence but strong depth dependence (2.6% in the lowest quartile at median 21,074 UMIs; 13.8% in the highest quartile at 133,551 UMIs), consistent with stochastic capture of rare transcripts in deeply sequenced cells rather than constitutive expression. At library sizes equivalent to typical astrocytes (∼4,000–10,000 UMIs), SLC13A5 detection in L5 ET neurons would approach background levels. All remaining 22 subclasses showed prevalence below 2.1% regardless of sequencing depth (Figure 1E). This cell-type specificity, visible across all 24 subclasses in the dot plot, contrasts with the canonical description of NaCT as a neuronal transporter [**?**] and is a primary finding of this study. Among the six transcriptomically defined astrocyte supertypes, *SLC13A5* expression was concentrated in Astro 2 (24.0% prevalence) and nearly absent from Astro 3 (0.87%; Figure 1F). The three other supertypes with sufficient nuclei for analysis (Astro 1, Astro 5, Astro 6) showed intermediate prevalence values ranging from approximately 8% to 15%.

SLC13A3, encoding the sodium-dependent dicarboxylate transporter NaDC3/SDCT2 [**?**], showed astrocyte-enriched expression at markedly higher prevalence (∼57%) and was additionally detected across several non-astrocyte subclasses.

Of the citrate metabolism enzymes, *ACO1*, *ACO2*, and *IDH1*/*IDH2* showed broad expression across multiple subclasses, while *SLC25A1* and *ACLY* showed enrichment in both astrocytes and neurons, at lower overall prevalence than *SLC13A3* (Figure 1E).

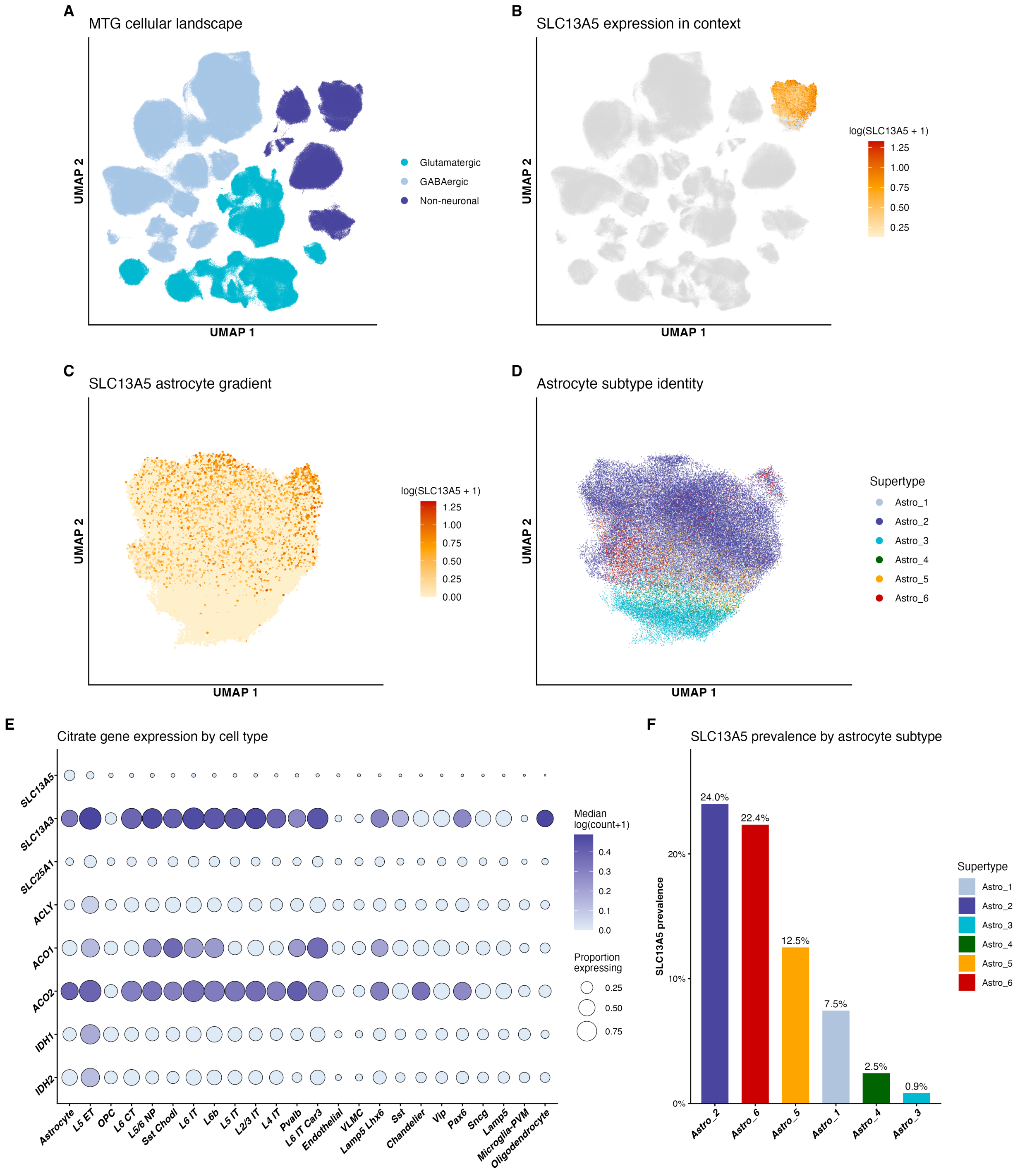

Figure 1: SLC13A5 expression landscape in the SEA-AD middle temporal gyrus. (A) UMAP of 1,378,211 nuclei by broad cell class. (B) SLC13A5-expressing astrocytes (orange-red) overlaid on the full UMAP. (C) SLC13A5 expression within the astrocyte cluster (zoomed). (D) Astrocyte supertype identity in the same zoomed view; Astro 2 is the primary SLC13A5-expressing population. (E) Proportion expressing (dot size) and median log-normalized count (dot color) for eight citrate metabolism genes across 24 cell subclasses. (F) SLC13A5 prevalence across the six astrocyte supertypes.

2.2 Overall SLC13A5 prevalence is stable across pseudoprogression

Cell-level Spearman correlation between pseudoprogression score and binary *SLC13A5* expression across 67,419 astrocyte nuclei yielded rho = -0.008 (FDR = 0.061; Figure 2C), indicating no meaningful monotonic trend. This null result was confirmed by logistic regression (pseudoprogression score OR not significant), by supertype-stratified analyses showing heterogeneous and partially opposing directional signals across supertypes, and by segmented regression identifying a weak non-monotonic shape with a potential inflection near pseudoprogression score 0.52 (SE = 0.10). At the donor level (n = 84), *SLC13A5* prevalence did not significantly correlate with mean pseudoprogression score (FDR $>$ 0.05; Figure 2D).

In contrast, *SLC13A3*, *ACO1*, *IDH2*, and *ACO2* all showed statistically significant negative cell-level correlations with pseudoprogression (FDR $<$ 0.001; Figure 2A), with *SLC13A3* showing the largest effect (rho = -0.080). At the donor level, *SLC13A3* exhibited a substantial negative correlation (rho = -0.362, FDR $<$ 0.05), confirming that the cell-level signal represents genuine inter-donor variability rather than pseudoreplication artifact.

2.3 Astrocyte subtype composition shifts underlie apparent SLC13A5 stability

Astrocyte supertype proportions changed significantly across pseudoprogression quintiles (Figure 2B). The Astro 2 proportion increased with pseudoprogression (Spearman rho = +0.345, FDR $<$ 0.001), while Astro 3 proportion declined (rho = -0.393, FDR $<$ 0.001). Because Astro 2 carries 24.0% *SLC13A5* prevalence and Astro 3 carries only 0.87%, this compositional shift would be expected to increase overall SLC13A5 prevalence; the expanding subtype expresses the gene at roughly 28-fold higher rates than the contracting one. That the overall prevalence remains stable therefore requires explanation. A concurrent within-Astro 2 transcriptional decline partially offsets the compositional upward pressure: cell-level Spearman correlation between pseudoprogression score and binary *SLC13A5* expression within Astro 2 nuclei yields rho = -0.043 (FDR $<$ 0.05). This effect is small in absolute magnitude and detectable only because of the large n, not because it is biologically large. The net result of these two opposing forces, compositional expansion of the high-expressing subtype and within-subtype transcriptional attenuation, is an apparent stability in overall *SLC13A5* prevalence that conceals a two-layer reorganization of the astrocytic compartment.

2.4 Astro 3 is an A1-reactive subtype; Astro 2 is homeostatic

To characterize the functional identities of these two supertypes, we extracted eight canonical reactive astrocyte markers (Figure 3A). Astro 3 showed 6.5-fold higher *C3* prevalence (10.4% vs.~1.6%; BH-corrected Wilcoxon $p < 0.001$) and 4.2-fold higher *CD44* prevalence (23.3% vs.~5.5%; $p < 0.001$) compared to Astro 2. *C3* and *CD44* are hallmarks of the A1/neurotoxic reactive state induced by microglial IL-1α, TNF, and C1q signaling [?]. In contrast, the pan-astrocyte marker *ALDH1L1* was modestly higher in Astro 2 (25.6% vs.~21.0%; $p < 0.001$), and *AQP4* prevalence was statistically indistinguishable between subtypes (25.5% vs.~24.2%; ns), confirming both populations as bona fide astrocytes [?]. Thus, Astro 3 represents an A1-like reactive subtype, while Astro 2 represents a more homeostatic population [?] that is the primary carrier of *SLC13A5*/NaCT expression. Accordingly, donor-level *SLC13A5* prevalence was substantially higher in Astro 2 than in Astro 3 nuclei across all 84 donors (Figure 3B).

2.5 Co-expression of citrate metabolism genes in SLC13A5+ nuclei

Phi coefficient analysis revealed weak but positive pairwise co-expression among all eight citrate metabolism genes (phi range: 0.01–0.15). *SLC13A5+* nuclei showed 2- to 3-fold enrichment for co-expression of *SLC13A3*, *ACO1*, and *ACLY* compared to *SLC13A5-* nuclei, suggesting that a subset of astrocytes maintains coordinated activity across the citrate import-to-utilization pathway (Figure 2E,F). The highest phi values were observed between metabolic enzymes in the same pathway segment (*SLC25A1–ACLY* and *ACO1–ACO2*), consistent with shared transcriptional regulation.

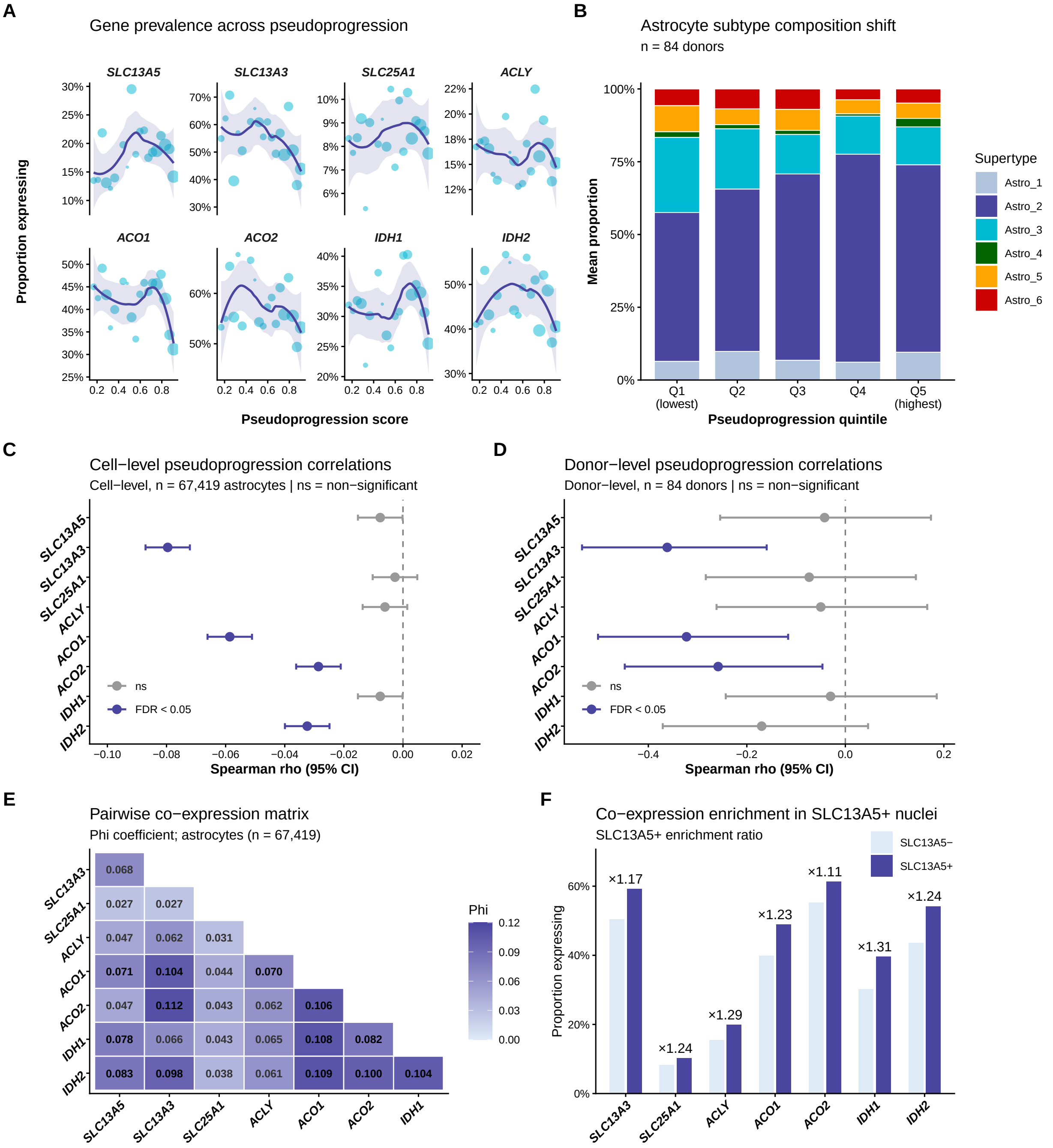

Figure 2: Pseudoprogression trajectories and co-expression architecture of citrate metabolism genes. (A) LOESS-smoothed prevalence trajectories for eight genes across pseudoprogression (20 bins; shading = 95% CI). (B) Astrocyte supertype composition per pseudoprogression quintile (n = 84 donors); Astro 2 expands and Astro 3 contracts. (C) Cell-level Spearman correlations between pseudoprogression score and binary gene expression (n = 67,419 astrocyte nuclei). (D) Donor-level Spearman correlations using aggregated prevalence (n = 84 donors). (E) Phi coefficient matrix for pairwise binary co-expression. (F) Proportion of each gene expressed in SLC13A5+ versus SLC13A5- nuclei; enrichment ratios shown above SLC13A5+ bars.

2.6 SLC13A5 and citrate gene expression correlate with neuropathological burden

At the donor level, *SLC13A5* prevalence in Astro 2 nuclei showed a modest negative association with Braak stage that did not survive BH correction (rho = -0.094, ns; Figure 3C), and negatively correlated with Thal phase (rho = -0.241, p = 0.027) but not with CERAD score (rho = -0.161, ns), suggesting that NaCT expression tracks the progressive spatial spread of amyloid pathology rather than local neuritic plaque density. Thal phase reflects the sequential propagation of amyloid-β deposits from allocortical to neocortical regions [?], whereas CERAD quantifies the density of neuritic plaques at the local sampling site [?]; their dissociation here indicates that the regional extent of amyloid spread, rather than its local intensity at the MTG sampling site, is the pathological variable most closely coupled to *SLC13A5* expression in Astro 2.

Sex-stratified analyses revealed that the Braak–SLC13A5 association was driven primarily by male donors (rho = -0.421, p = 0.015, n = 33), while female donors showed no significant correlation (rho = -0.153, p = 0.284, n = 51). A linear interaction model did not detect Braak × sex interaction (β = -0.028, p = 0.148), likely reflecting limited power given the male subsample size. This pattern should be interpreted cautiously but warrants investigation in larger cohorts with balanced sex representation.

SLC13A3 and *ACO1* showed more robust correlations across both pathological measures: *SLC13A3* with Thal (rho = -0.310, BH-adj. p = 0.009) and CERAD (rho = -0.342, p = 0.006); *ACO1* with Thal (rho = -0.307, p = 0.009) and CERAD (rho = -0.349, p = 0.006). Confound checks confirmed that neither age at death (Spearman rho = 0.106, p = 0.337) nor post-mortem interval (rho = 0.119, p = 0.282) meaningfully predicted *SLC13A5* prevalence in Astro 2 nuclei (Figure 3E). Cognitive status (No dementia vs.~Dementia) was not significantly associated with expression of any of the eight genes after BH correction.

2.7 APOE4 genotype is associated with lower SLC13A5 prevalence in Astro 2

APOE4 carriers (n = 23) showed lower median *SLC13A5* prevalence in Astro 2 nuclei compared to APOE4- donors (n = 61; median 17.6% vs.~25.9%; Wilcoxon p = 0.025; Figure 3D). This difference was not statistically significant across all astrocyte nuclei combined (p = 0.054), consistent with signal dilution by Astro 3 nuclei where *SLC13A5* is nearly absent. In multivariate OLS regression adjusting for pseudoprogression, age, and sex, the APOE4 coefficient did not reach significance (p = 0.484; Figure 3E; R^2 = 0.065), indicating that the association detected by the non-parametric test is not robust to regression modeling of the skewed outcome distribution. *ACO1* prevalence in Astro 2 showed a similarly borderline APOE4 association (Wilcoxon p = 0.025), while *SLC13A3* and *ACO2* did not differ by APOE4 status.

2.8 SLC13A5 expression does not distinguish cognitively resilient from expected-AD donors

To test whether higher NaCT expression might confer cognitive resilience against equivalent tau pathology burden, we compared *SLC13A5* prevalence in Astro 2 between donors with high Braak stage (IV–VI) who retained cognitive function (Resilient; n = 34) versus those with dementia (Expected AD; n = 38). Median Astro 2 *SLC13A5* prevalence was 23.0% in Resilient and 22.7% in Expected AD donors (Wilcoxon p = 0.888; Figure 3F). Kruskal-Wallis test across all three resilience groups (including Low pathology; n = 12) was also non-significant. The cognitive

resilience hypothesis for NaCT-mediated citrate transport in MTG astrocytes is not supported by this dataset. These findings are specific to Astro 2 nuclei in the middle temporal gyrus; whether SLC13A5 expression in other brain regions, particularly those where neuronal expression may be more prominent, contributes to cognitive resilience mechanisms cannot be excluded.

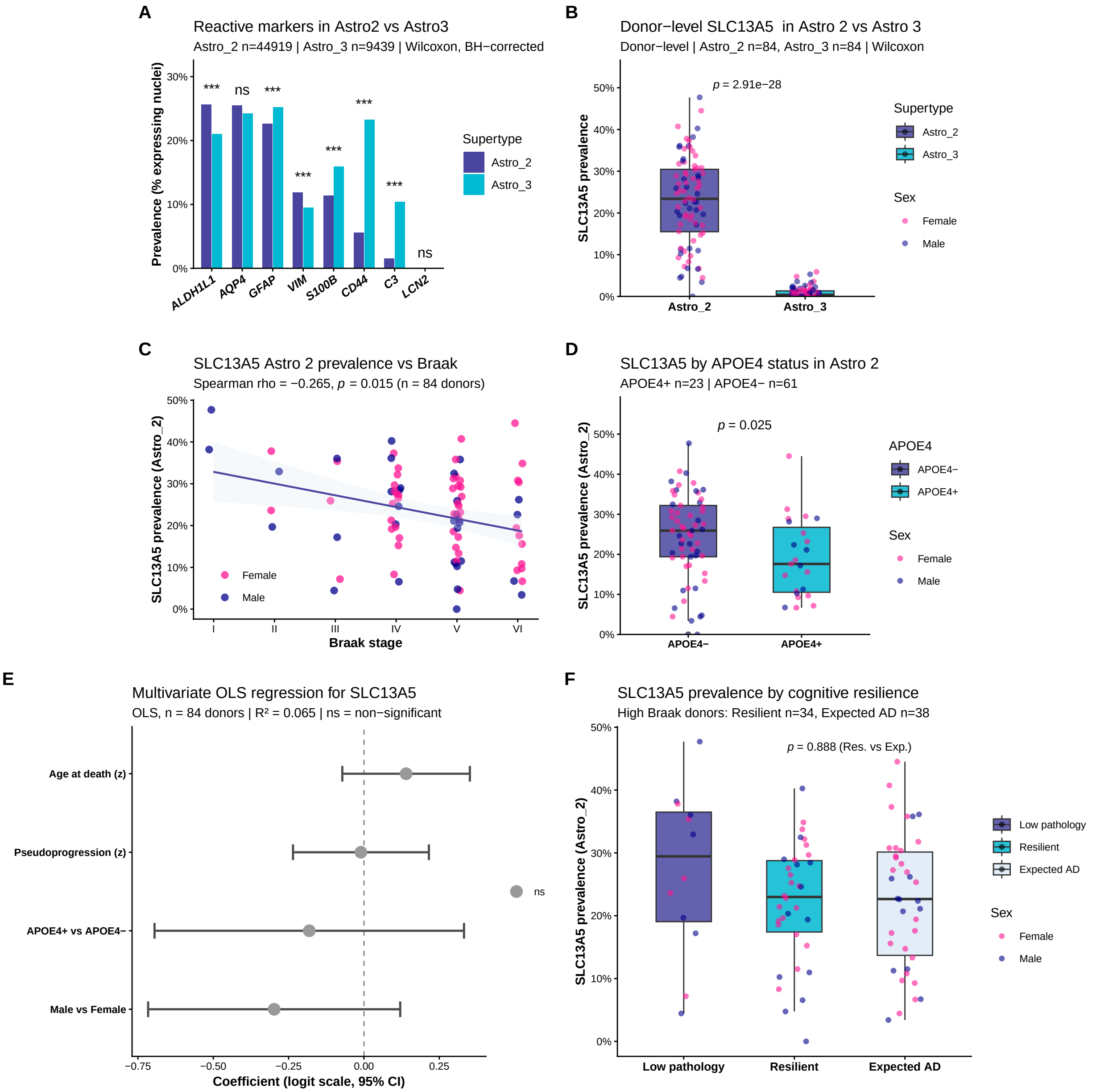

Figure 3: Neuropathological, genetic, and subtype-specific determinants of astrocyte SLC13A5 expression. (A) Reactive astrocyte marker prevalence in Astro 2 versus Astro 3 (Wilcoxon, BH-corrected; *** FDR < 0.001). (B) Donor-level SLC13A5 prevalence in Astro 2 versus Astro 3. (C) SLC13A5 prevalence in Astro 2 nuclei versus Braak stage (Spearman correlation; each point = one donor, colored by sex; regression line with 95% CI). (D) SLC13A5 prevalence in Astro 2 by APOE4 genotype (Wilcoxon rank-sum). (E) Multivariate OLS regression coefficients for predictors of logit-transformed SLC13A5 prevalence ($n = 84$ donors). (F) SLC13A5 prevalence across cognitive resilience groups (Low pathology: Braak I–III; Resilient: Braak IV–VI + No dementia; Expected AD: Braak IV–VI + Dementia).

3 Supplementary material

3.1 Supplementary Table 1. Cell-level Spearman correlations for all eight citrate metabolism genes across pseudoprogression in astrocytes (n = 67,419 nuclei).

Table 1: Supplementary Table 1. Cell-level Spearman correlations (pseudoprogression vs. binary expression, astrocyte nuclei).

Gene	Spearman rho	p-value	FDR	Significance	n nuclei
SLC13A5	-0.008	0.0449	0.0609	ns	67419
SLC13A3	-0.080	0.0000	0.0000	***	67419
SLC25A1	-0.003	0.4890	0.4890	ns	67419
ACLY	-0.006	0.1160	0.1330	ns	67419
ACO1	-0.059	0.0000	0.0000	***	67419
ACO2	-0.029	0.0000	0.0000	***	67419
IDH1	-0.008	0.0456	0.0609	ns	67419
IDH2	-0.032	0.0000	0.0000	***	67419

3.2 Supplementary Table 2. Donor-level Spearman correlations for all eight genes.

Table 2: Supplementary Table 2. Donor-level Spearman correlations (mean pseudoprogression vs. bulk astrocyte prevalence, n = 84 donors).

Gene	Spearman rho	p-value	FDR	Significance	n donors
SLC13A5	-0.042	0.704000	0.78600	ns	84
SLC13A3	-0.362	0.000723	0.00578	**	84
SLC25A1	-0.073	0.507000	0.78600	ns	84
ACLY	-0.050	0.652000	0.78600	ns	84
ACO1	-0.322	0.002770	0.01110	*	84
ACO2	-0.258	0.017700	0.04710	*	84
IDH1	-0.030	0.786000	0.78600	ns	84
IDH2	-0.170	0.122000	0.24500	ns	84

3.3 Supplementary Table 3. Neuropathological correlations for all eight citrate metabolism genes.

Table 3: Supplementary Table 3. Neuropathological correlations for citrate gene prevalences. Braak correlations use donor-level prevalence across all astrocytes (n = 84 donors). Thal and CERAD correlations are Astro 2-specific (SLC13A5, SLC13A3, ACO1, ACO2 only).

Gene	Pathology measure	Spearman rho	p-value	FDR	Sig.	n donors
ACLY	Braak stage	-0.086	4.38e-01	0.438000	ns	84
ACO1	Braak stage	-0.430	4.51e-05	0.000361	***	84
ACO1	CERAD score	-0.349	1.13e-03	0.005760	**	84
ACO1	Thal	-0.307	4.49e-03	0.008970	**	84
ACO2	Braak stage	-0.198	7.05e-02	0.113000	ns	84
ACO2	CERAD score	-0.076	4.92e-01	0.492000	ns	84
ACO2	Thal	-0.156	1.57e-01	0.179000	ns	84
IDH1	Braak stage	-0.212	5.29e-02	0.106000	ns	84
IDH2	Braak stage	-0.310	4.17e-03	0.016100	*	84
SLC13A3	Braak stage	-0.297	6.04e-03	0.016100	*	84
SLC13A3	CERAD score	-0.342	1.44e-03	0.005760	**	84
SLC13A3	Thal	-0.310	4.14e-03	0.008970	**	84
SLC13A5	Braak stage	-0.094	3.95e-01	0.438000	ns	84
SLC13A5	CERAD score	-0.161	1.42e-01	0.179000	ns	84
SLC13A5	Thal	-0.241	2.73e-02	0.043700	*	84
SLC25A1	Braak stage	-0.091	4.08e-01	0.438000	ns	84

3.4 Supplementary Table 4. Reactive astrocyte marker comparison: Astro 2 vs. Astro 3.

Table 4: Supplementary Table 4. Wilcoxon rank-sum comparison of reactive astrocyte marker prevalences between Astro 2 and Astro 3 supertypes (astrocyte nuclei, BH-corrected).

Marker	Prevalence Astro 2	Prevalence Astro 3	log2FC (A2/A3)	p-value	FDR	Sig.
GFAP	0.226	0.252	-0.16	0.000	0.000	***
VIM	0.119	0.095	0.33	0.000	0.000	***
C3	0.016	0.104	-2.74	0.000	0.000	***
ALDH1L1	0.256	0.210	0.28	0.000	0.000	***
AQP4	0.255	0.242	0.07	0.363	0.415	ns
S100B	0.114	0.159	-0.49	0.000	0.000	***
CD44	0.055	0.233	-2.07	0.000	0.000	***
LCN2	0.000	0.000	Inf	0.517	0.517	ns

3.5 Supplementary Table 5. SLC13A5 expression correlations with pseudoprogression per astrocyte supertype.

Table 5: Supplementary Table 5. SLC13A5 Spearman correlations with pseudoprogression per astrocyte supertype. Prevalence: binary expression vs. pseudo-score across all nuclei in each supertype (Astro 4 excluded; < 50 expressing cells). Intensity: log1p-transformed counts among SLC13A5-expressing nuclei only (Astro 3 and Astro 4 additionally excluded due to < 100 expressing cells).

Supertype	Measure	n nuclei	n expressing	Spearman rho	p-value	FDR
Astro_1	Intensity (SLC13A5+ only)	399	399	-0.004	9.30e-01	9.30e-01
Astro_1	Prevalence (binary)	5335	399	-0.059	1.55e-05	3.87e-05
Astro_2	Intensity (SLC13A5+ only)	10800	10800	0.042	1.08e-05	4.31e-05
Astro_2	Prevalence (binary)	44919	10800	-0.043	0.00e+00	0.00e+00
Astro_3	Prevalence (binary)	9439	82	0.015	1.34e-01	1.34e-01
Astro_5	Intensity (SLC13A5+ only)	452	452	-0.055	2.45e-01	3.26e-01
Astro_5	Prevalence (binary)	3603	452	-0.037	2.83e-02	3.54e-02
Astro_6	Intensity (SLC13A5+ only)	732	732	0.046	2.10e-01	3.26e-01
Astro_6	Prevalence (binary)	3271	732	0.043	1.29e-02	2.15e-02

3.6 Supplementary Table 6. Segmented regression of SLC13A5 astrocyte prevalence across pseudoprogression.

Table 6: Supplementary Table 6. Segmented ordinary least-squares regression of SLC13A5 astrocyte prevalence across 20 equal-width pseudoprogression bins (n = 19 bins with data; weighted by nuclei per bin). Davies test evaluates whether the breakpoint improves fit over a linear model.

Parameter	Value
Breakpoint (pseudo-score)	0.5195
Breakpoint SE	0.0992
Slope before breakpoint (per unit pseudo-score)	0.3319
Slope after breakpoint	-0.1940
Adjusted R^2	0.4143
Davies test p-value (breakpoint significance)	0.0392

3.7 Supplementary Figure S1. Sex-stratified SLC13A5 and citrate metabolism gene expression trajectories across pseudoprogression.

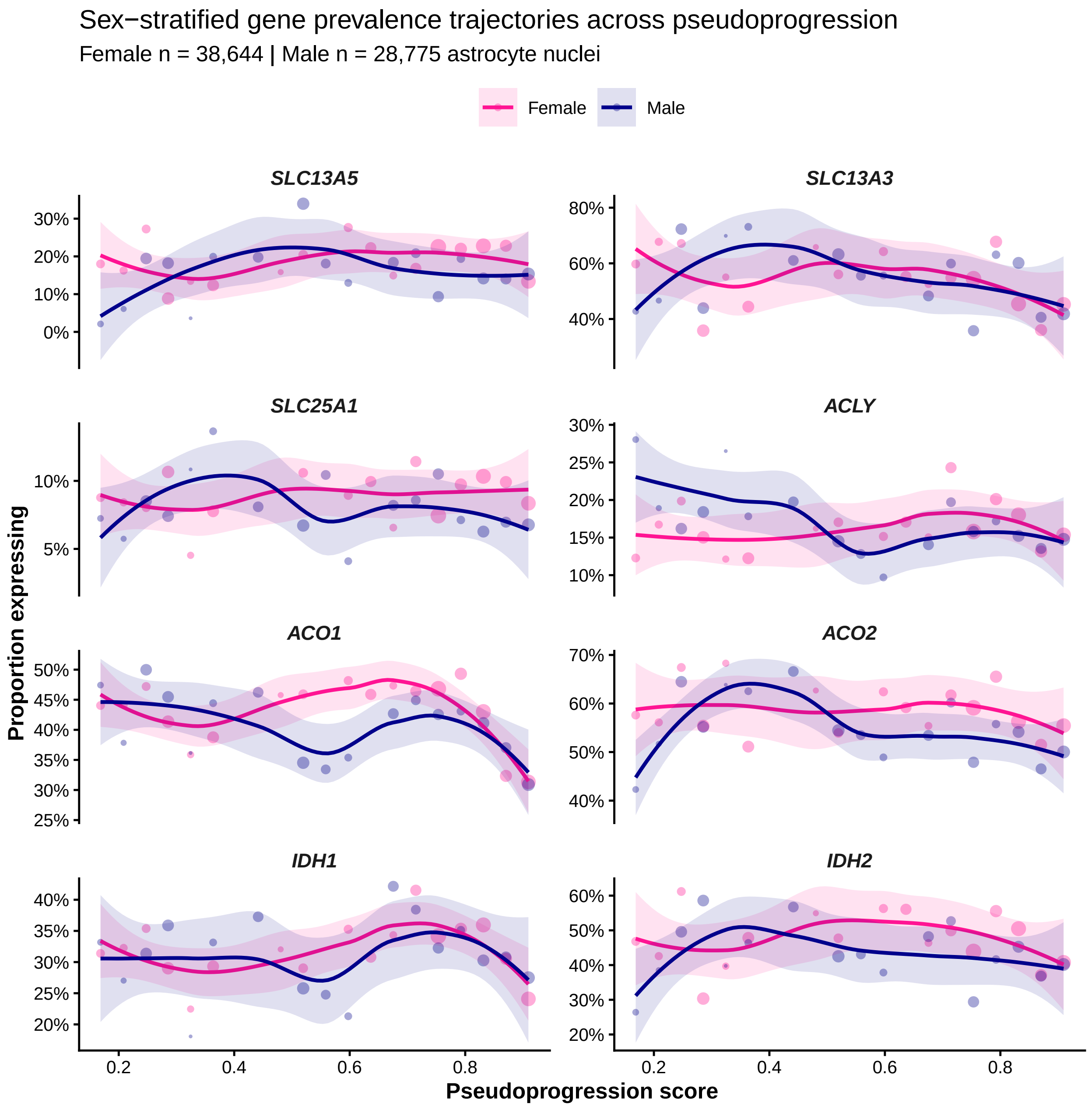

3.8 Supplementary Figure S2. Cognitive status comparison across all eight citrate metabolism genes.

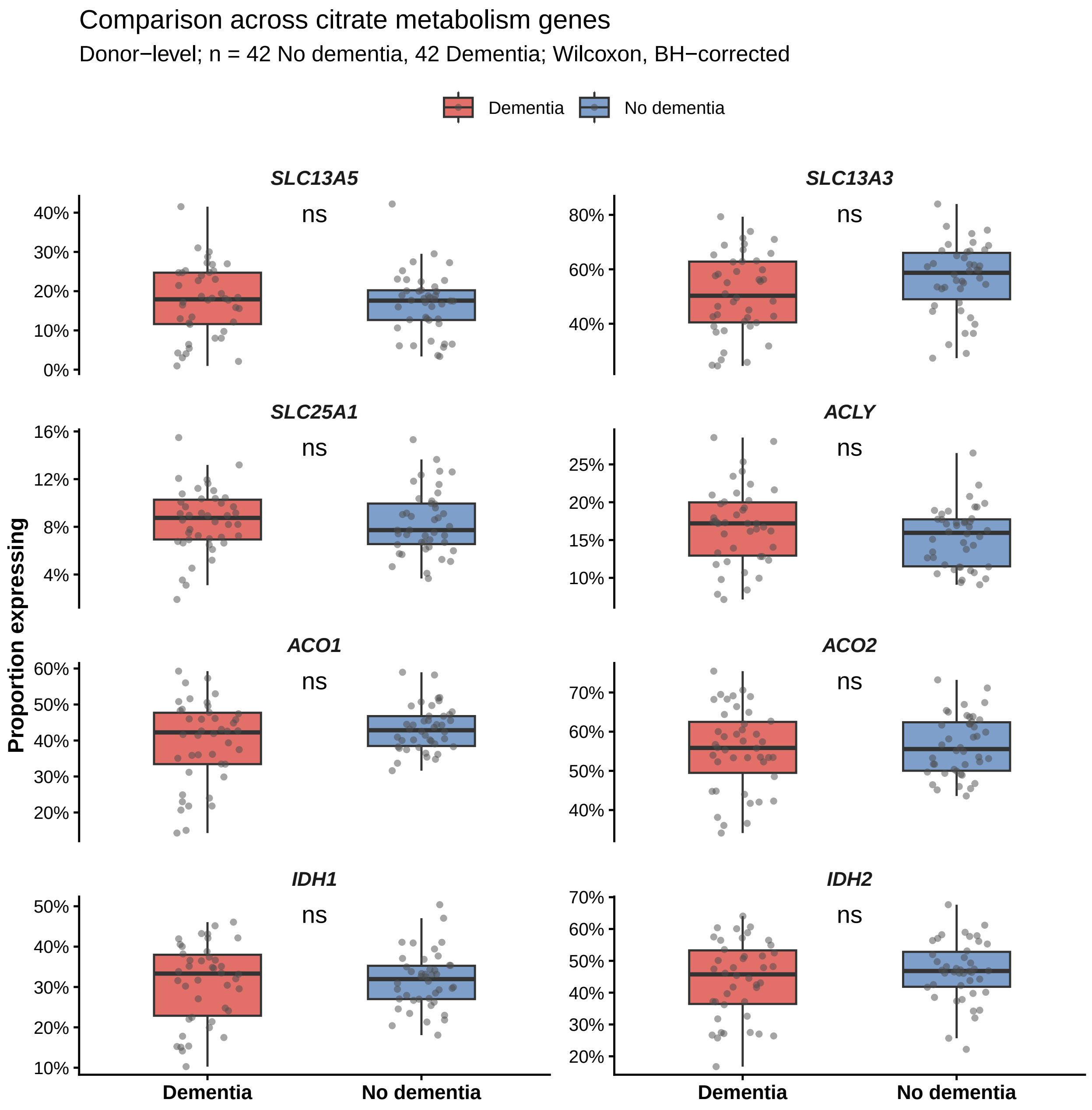

3.9 Supplementary Figure S3. SLC13A5 astrocyte trajectory breakpoint and per-supertype pseudoprogression correlations.

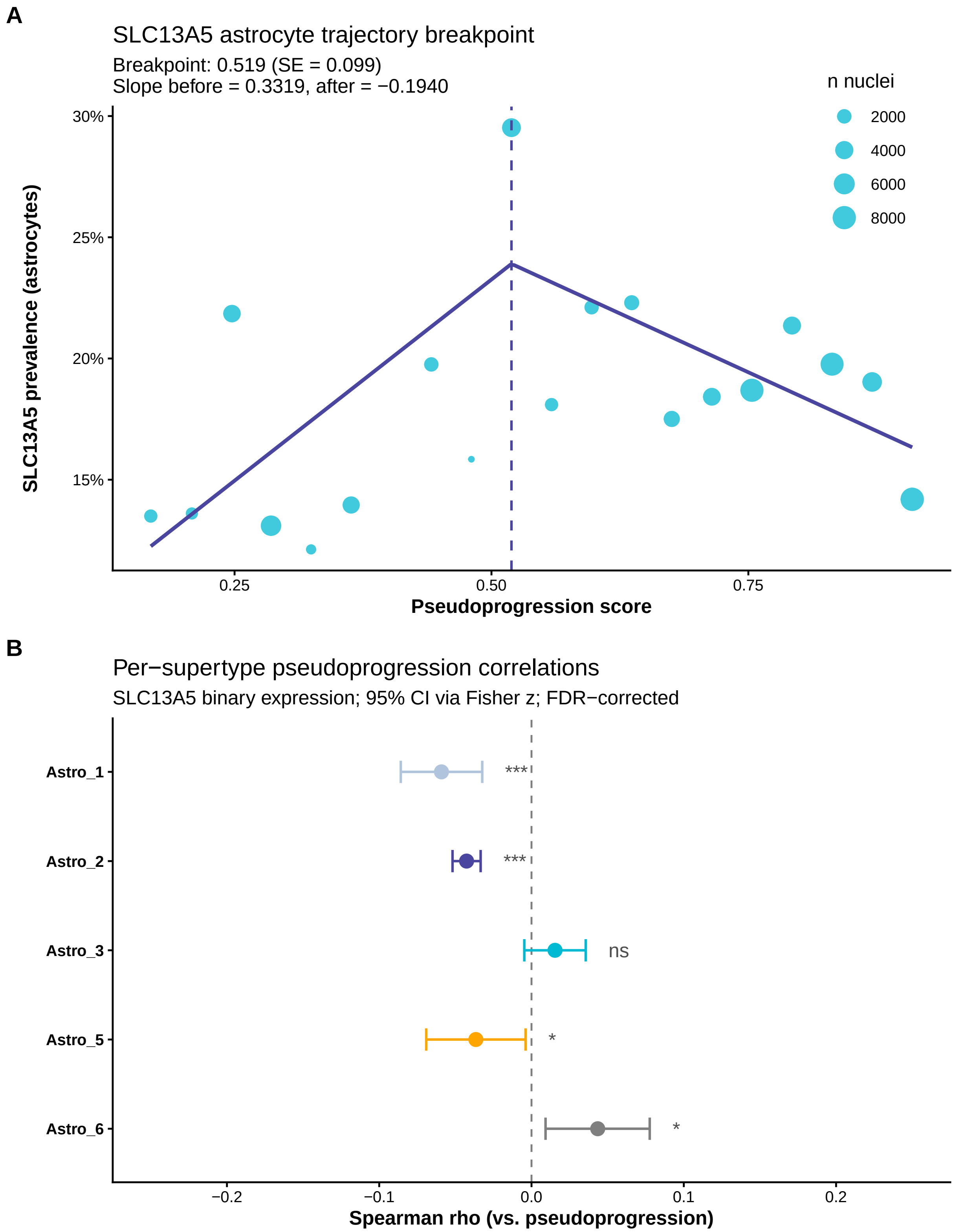

3.10 Supplementary Figure S4. Neuropathological and genetic associations across all eight citrate metabolism genes.

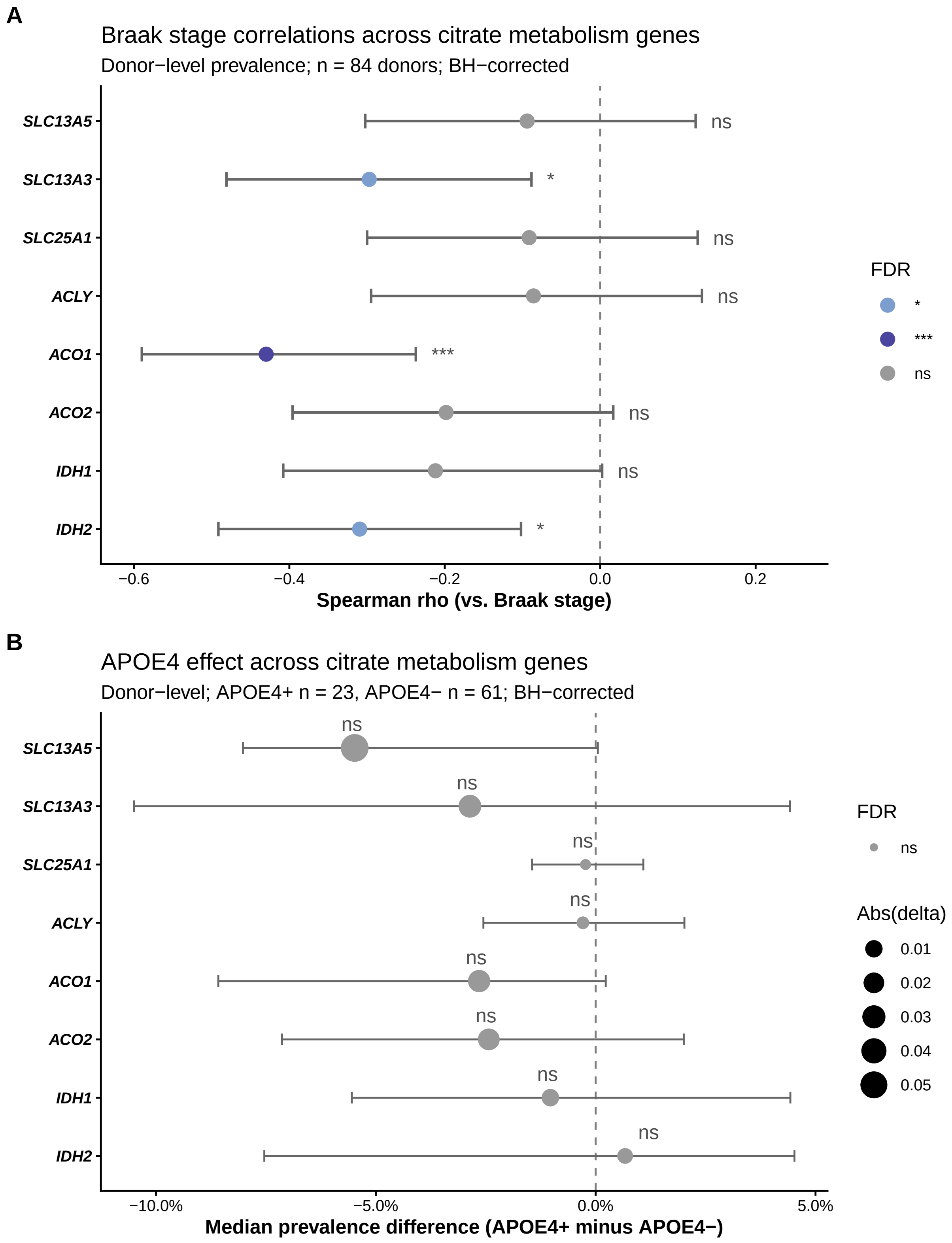

Supplement: Supplementary file 1 [file cimb-48-00691-s001.zip › Supplementary_files_cimb_final/NEC03V02D0526_MDPI_CSR.pdf]
