# Supplementary figures and images for: Astrocyte Subtype-Specific Expression of the Sodium-Coupled Citrate Transporter SLC13A5 and Citrate Metabolism Genes Across Alzheimer’s Disease Pseudoprogression: A Single-Nucleus RNA Sequencing Analysis of the Human Middle Temporal Gyrus
Source: Curr Issues Mol Biol. 2026 Jul 5;48(7):691. doi: 10.3390/cimb48070691 (PMC13407191; doi:10.3390/cimb48070691)

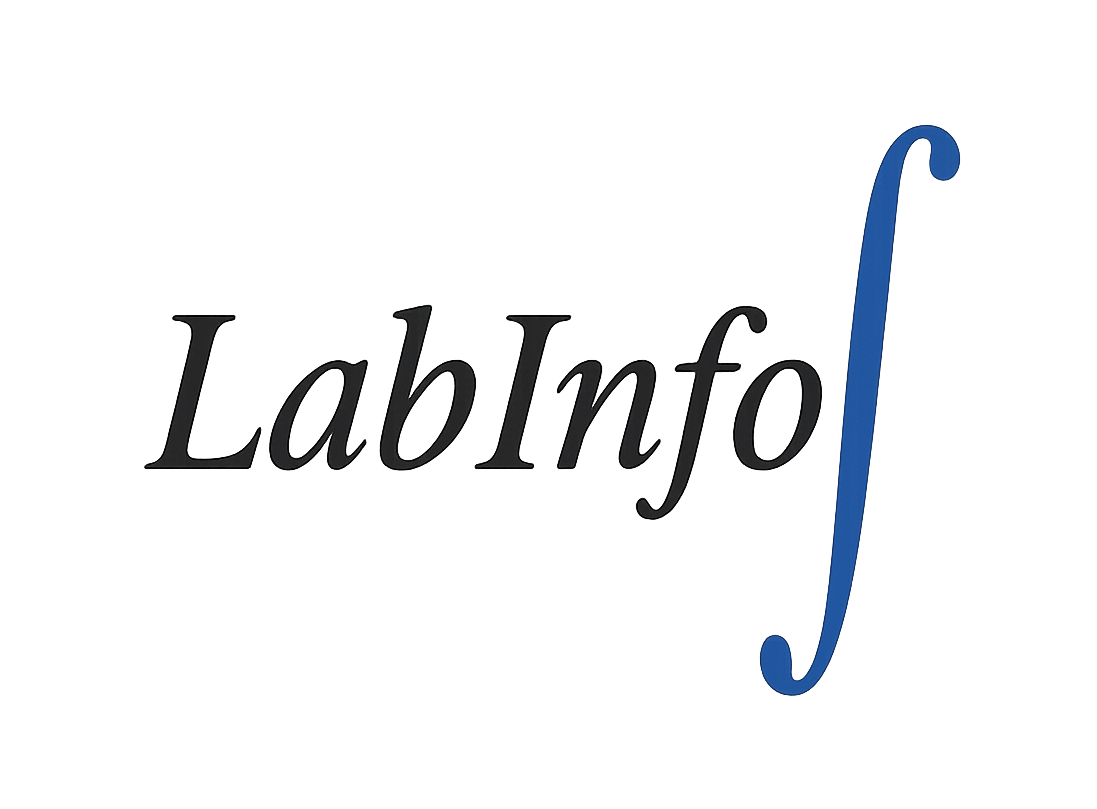

Supplement: Supplementary file 1 [file cimb-48-00691-s001.zip › Supplementary_files_cimb_final/NEC_logo.png]
